# Long QT Syndrome With Drugs Used in the Management of Arrhythmias: A Systematic Review

**DOI:** 10.7759/cureus.65857

**Published:** 2024-07-31

**Authors:** Shenel A Khan, Soniya Emmanuel, Vivig Shantha Kumar, Resheek Nerella, Basim Shaman Ameen, Dev Patel, Jabez David John, Ranita Bodepudi, Saniya Seher, Sai Sri Penumetcha

**Affiliations:** 1 General Medicine, California Institute of Behavioral Neurosciences & Psychology, Fairfield, USA

**Keywords:** cardiac conduction disorder, torsades de pointes (tdp), drug therapy, arrhythmia, long qt syndrome

## Abstract

Long QT syndrome (LQTS) is a severe cardiac disorder characterized by an abnormally prolonged QTc interval on an electrocardiogram (ECG), which can result in life-threatening irregular heart rhythms. The use of certain medications, particularly anti-arrhythmic drugs such as quinidine, sotalol, and amiodarone, can lead to acquired LQTS by prolonging the QT interval through the inhibition of specific ion channels responsible for heart repolarization, which may present symptoms like fainting, seizures, and sudden cardiac arrest. This systematic review, conducted following the Preferred Reporting Items for Systematic Reviews and Meta-Analyses (PRISMA) 2020 guidelines, focused on analyzing the association between Long QT syndrome and drugs utilized for managing arrhythmias, involving a thorough examination of six selected studies from an initial pool of 68 articles. It was found that antiarrhythmic drugs such as amiodarone, sotalol, dofetilide, procainamide, quinidine, and flecainide have the potential to cause QT prolongation as a side effect, which is often influenced by factors including dosage, coexisting medical conditions, electrolyte imbalances, and other risk factors. Prolonged QT interval significantly elevates the risk of a life-threatening arrhythmia called torsade de pointes. The management of this side effect typically involves reducing the medication dosage or discontinuing it altogether and, in some cases, employing selective beta blockers. However, further research is essential to improve the understanding and implementation of strategies to prevent and manage QT prolongation caused by antiarrhythmic drugs. Additional clinical studies are warranted to enhance knowledge and provide comprehensive guidelines to healthcare practitioners regarding the appropriate use of these medications. Close monitoring of the QT interval is recommended for patients receiving anti-arrhythmic therapy, and consideration should be given to patient-specific risk factors for LQTS, including age, sex, and electrolyte imbalances.

## Introduction and background

Depolarization and repolarization are fundamental aspects of the cardiac electrical system. One can observe distinctive P waves, QRS complex, and T waves on an electrocardiogram (ECG) to visualize them. In clinical practice, the QT interval, which spans from the Q wave to the T wave on an electrocardiogram (ECG), serves as an indicator of both ventricular depolarization and repolarization. Specifically, the QRS complex signifies ventricular depolarization, while the JT interval reflects ventricular repolarization. Together, the QRS complex and JT interval contribute to the overall QT interval measurement [1,2].

A prolongation of the QT interval on an electrocardiogram indicates the presence of the Long QT Syndrome (LQTS), which is a disorder affecting cardiac repolarization. A prolonged QT interval can be caused by a decrease in the overall current responsible for repolarization, which could be due to a decrease in outward K+ currents, specifically the delayed rectifier K+ current (IKr), or an increase in inward late Na+ current (INaL). In most cases, drug-induced LQTS arises from the blockage of the rapidly delayed rectifier potassium channel (IKr).

Long QT Syndrome (LQTS) can result in various severe symptoms such as palpitations, syncope, seizures, and sudden cardiac death. In addition, the presence of this syndrome increases the likelihood of developing torsades de pointes, a dangerous cardiac arrhythmia that manifests as polymorphic ventricular tachycardia and can be life-threatening. This arrhythmia is characterized by a twisting pattern on the electrocardiogram and may spontaneously terminate and revert to sinus rhythm, or it may deteriorate into ventricular fibrillation, a type of cardiac arrest that can be fatal if not promptly treated. Therefore, individuals with LQTS require close monitoring to detect any abnormal heart rhythms, and prompt intervention should be taken in case of any life-threatening events.

Several formulae are available to calculate the QTc interval, including Bazett, Fridericia, and Framingham [3-5]. Among these, the most commonly utilized formula is Bazett's, which entails the division of the QT interval by the square root of the R-R interval. This method has been widely adopted in clinical practice due to its simplicity and ease of calculation.

Prolongation of the QT interval can be categorized into two primary types: congenital causes and acquired causes. Acquired causes are more commonly observed than congenital causes. Congenital causes are linked to more than 15 mutations in ion channels such as potassium, calcium, or sodium channels [6].

In contrast, acquired QT interval prolongation can result from drug therapy or electrolyte imbalances that affect these ion channels. Due to the vast array of medications that can cause QT interval prolongation, pharmacological agents are the primary contributing factor to acquired LQTS. Therefore, it is essential to closely monitor patients on medication that can affect the QT interval and to manage any electrolyte disturbances promptly to prevent the development of LQTS. Table 1* *shows several medications that can cause prolongation of the QT interval.

**Table 1:** Medications known for their potential to induce prolongation of the QT interval

Anti psychotics	Anti arrhythmics	Antibiotics	Anti depressants	Other medications
Haloperidol	Amiodarone	Macrolides	Amitryptiline	Sumatriptan
Ziprasidone	Sotalol	Fluoroquinolones	Imipramine	Ondansetron
Quetiapine	Dofetilide	-	Citalopram	Cisapride
Thioridazine	Procainamide	-	-	-
Olanzapine	Quinidine	-	-	-
Risperidone	Flecainide	-	-	-

Our research endeavor aimed to perform a comprehensive and systematic review of the association between Long QT Syndrome (LQTS) and medications that are commonly utilized in the management of arrhythmia. Our primary objective was to identify the specific drug interactions that that might play a role in the prolongation of the QT interval, which is a well-known risk factor for life-threatening arrhythmias. By systematically reviewing relevant studies and analyzing the available data, we aimed to shed new light on the potential mechanisms underlying drug-induced QT prolongation and to identify the most significant contributors to this phenomenon. Our findings may help to inform clinical decision-making and optimize medication management in patients with LQTS or those at risk for drug-induced QT prolongation.

## Review

Methodology

Preferred Reporting Items for Systematic Reviews and Meta-Analyses (PRISMA) guidelines of 2020 were followed for the planning and execution of this systematic review.

Eligibility Criteria

We established particular inclusion and exclusion criteria to ensure the selection of pertinent and high-quality articles for our study. Our inclusion criteria consisted of articles published in English within the past ten years and available in full-text format. We included case reports, observational studies, systematic reviews, and comparative review articles pertaining to long QT syndrome, arrhythmia, drug therapy, and human subjects aged 19 years or older.

Our exclusion criteria were based on the methodological quality of the articles. We excluded paid articles, articles that related to patients under 19 years of age, and articles that focused on individuals with pacemakers. By adhering to these criteria, we aimed to ensure that the articles we included in our study were of high quality and could provide relevant insights into our research question.

Databases and Search Strategy

We conducted a systematic search using PubMed to identify relevant articles for our study. The selection of search terms was based on both previously used keywords and Medical Subject Headings (MeSH) to ensure a comprehensive and relevant search. To facilitate the reproducibility of our search process, we developed a search strategy, which is detailed in the table below. By using a systematic approach, we aimed to identify all potentially relevant articles and reduce the risk of bias in our study. Our search strategy enabled us to identify a comprehensive set of articles that met our inclusion criteria, providing a robust evidence base for our analysis. Table 2 shows the database search results for Long QT syndrome, Arrhythmia, and Drug Therapy. For the purpose of removing duplicates, all references were collected using Microsoft Excel. After reviewing the abstracts, irrelevant studies were removed from the records.

**Table 2:** Database search results for Long QT syndrome, arrhythmia, and drug Therapy Sources: PubMed, PubMed Central, and Medline (Last 10 Years).

Databases	Keywords	Search strategy	Filters	Search results
PubMed, PubMed Central and Medline	Long QT syndrome, arrhythmia and drug therapy	Long QT syndrome OR ( "Long QT Syndrome/analysis"[Majr] OR "Long QT Syndrome/complications"[Majr] OR "Long QT Syndrome/drug therapy"[Majr] ) AND Arrhythmia OR ( "Arrhythmias, Cardiac/complications"[Majr] OR "Arrhythmias, Cardiac/drug therapy"[Majr] ) AND Drug induced	Free papers, last 10 years, humans, English, Adults 19+	68

Results

Study Selection

**Figure 1:**
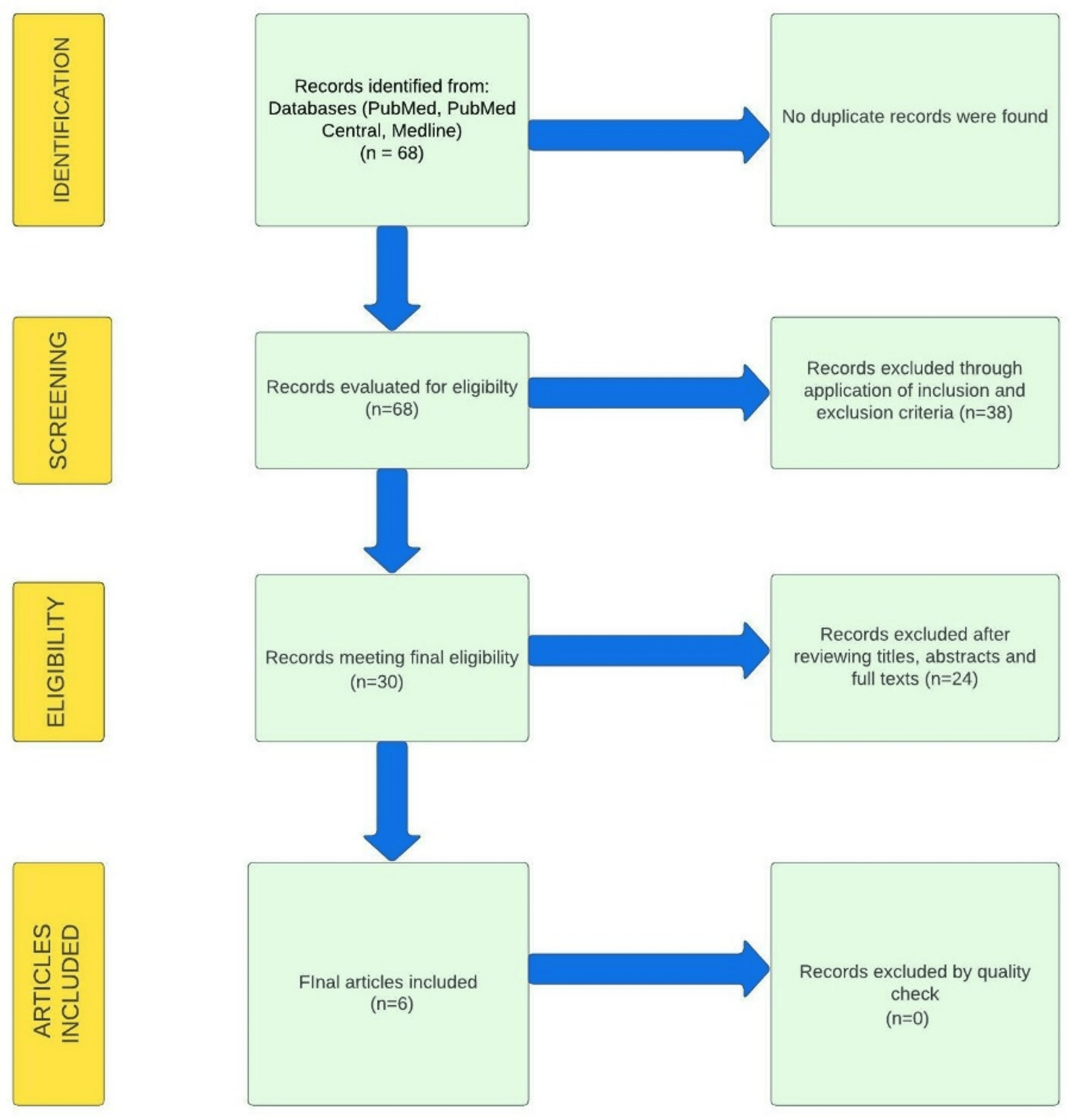
PRISMA 2020 flow diagram for this study PRISMA: Preferred reporting items for systematic reviews and meta-analysis [7]. The flow diagram effectively visualizes the literature and search strategy employed in this study.

Our search of the PubMed database yielded 68 potentially significant titles, of which 30 were retained for full-text review. From these, we selected six studies, including three case reports, one case series, one retrospective observational study, and one comparative observational study, based on a quality assessment using validated scales specific to each type of study design. We employed a systematic approach adhering to the PRISMA guidelines for study selection in our research process. The figure below illustrates the PRISMA flow diagram, which outlines the literature search strategy and study selection process. Figure 1 shows the PRISMA flow chart of this systematic review [7].

Quality Assessment/Risk of Bias in Individual Studies

We applied validated scales specific to each type of study design to evaluate the quality and bias risk of the publications included in our study. Two reviewers separately evaluated each paper, with disputes being resolved through discussion and, if necessary, consultation with a third reviewer. Using these validated scales, we aimed to assess the quality and risk of bias of the articles included in our study. This enabled us to identify the strengths and weaknesses of each study and to draw more reliable conclusions based on the most rigorous and high-quality evidence available.

We meticulously evaluated the quality of six validated articles through the utilization of two assessment tools: the JBI checklist (n=4) designed for case reports/case series and the New Castle Ottawa scale (n=2) intended for observational studies. Among the four case reports, one achieved a score of eight out of eight, while the remaining three scored seven out of eight on the JBI checklist. As for the two observational studies, both obtained a score of seven out of eight on the New Castle Ottawa scale. Subsequently, we included all six articles (n=6) that met high-quality criteria on both the JBI checklist and Newcastle-Ottawa checklist in our review.

Discussion

Our article focuses on investigating the association of long QT syndrome (LQTS) with anti-arrhythmic drugs. Normally, males have a QTc of 440 ms or less, whereas females have a QTc of 460 ms or less [8]. Although it can be challenging to determine what changes to the QT interval are clinically relevant, the FDA acknowledges that a time interval of more than 500 ms or a change of more than 60 ms can be categorised as prolonged.

Individuals with a prolonged QT interval are at risk of developing Torsades de Pointes (TdP), a potentially fatal arrhythmia. Several antiarrhythmic drugs, such as amiodarone, sotalol, dofetilide, procainamide, quinidine, and flecainide, can contribute to QT interval prolongation. These medications bind to the channels of the human ether-related gene (hERG), inhibiting potassium ion channel electrical conduction and thereby delaying the heart's repolarization. Managing drug-induced QT prolongation typically involves discontinuing the offending medication. Correction of any associated electrolyte imbalances is crucial to reduce the likelihood of TdP [9,10].

Amiodarone

Amiodarone, classified as a type III antiarrhythmic drug, is utilized for treating arrhythmias that occur in both the ventricles and the upper chambers of the heart. It can, however, also be proarrhythmic and is known to have a risk of prolonging QTc and causing TdP [11]. Amiodarone is commonly administered intravenously at a dose of 1,475 mg within 48 hours of treatment initiation and subsequently maintained at a daily dosage of 600 mg for a duration of days or weeks as required, for adult patients diagnosed with hemodynamically unstable ventricular fibrillation or ventricular tachycardia. Following this initial period, the medication is transitioned to oral administration. However, these practices may vary [12,13].

Takahashi et al. (2021) documented a case of an 83-year-old Japanese woman who exhibited symptoms of atrial fibrillation (AF) and was subsequently diagnosed with acute decompensated heart failure (ADHF). Intravenous administration of amiodarone to manage AF led to non-sustained TdP runs on day 14 of hospitalization, which was associated with amiodarone-induced QT interval prolongation. Therefore, treatment was continued with oral amiodarone hydrochloride at a reduced dose of 200 mg/day, and landiolol hydrochloride, a β1-superselective adrenergic antagonist, was administered to resolve TdP [14].

Mourad et al. conducted a comparative research study to investigate the potential effects of co-administration of voriconazole and amiodarone on QT interval prolongation, as well as any associated cardiac arrhythmias that may have resulted from such concomitant use [15].

Amiodarone is extensively metabolized in the liver via several cytochrome P450 pathways, including CYP3A4, and possesses a remarkably long half-life of 40-55 days. The concomitant administration of voriconazole, which is also a CYP3A4 inhibitor, may potentially lead to increased concentrations of amiodarone, thereby heightening the risk of adverse effects [16]. This research study comprised 46 adult patients who were admitted to a tertiary care hospital between 1st January 2005 and 31st December 2015, and who received concurrent systemic therapy with voriconazole and amiodarone for a minimum of two days. Baseline and follow-up electrocardiograms (ECGs) were obtained and analyzed for each patient.

In the subsequent analysis, it was found that the concomitant administration of voriconazole and amiodarone resulted in a statistically significant prolongation of the QTc interval by 49 ms, which was higher than the prolongation observed with amiodarone (+29 ms) or voriconazole (+26.2 ms) when used alone. The average increase in QTc interval observed with amiodarone alone was +29 ms [17], while that observed with voriconazole alone was +26.2 ms [18].

Sotalol/Dofetilide

Dofetilide and Sotalol are categorized as class III anti-arrhythmic drugs, which selectively impede the delayed rectifier K+ current (IKr), leading to an increase in the effective refractory period. These drugs, similar to other medications that impact potassium current, have the ability to lengthen the QT interval and provoke ventricular tachyarrhythmias, namely TdP. Hence, patients who are administered sotalol and dofetilide necessitate hospitalization to ensure proper monitoring and adjustment of doses [19].

In a retrospective study done by Ting et al. in 2020, adult patients admitted to a teaching hospital between June 1, 2015 and August 1, 2018, were observed, who were given sotalol or dofetilide [20]. This study looked at how sotalol and dofetilide were prescribed and assessed the safety results of both nonstandard and standard dosing regimens. A dose that exceeded the FDA-recommended dosage, taking into account the QTc and estimated creatinine clearance, or both, was defined as nonstandard dosing in the study [20]. The study analyzed 379 patients who were prescribed either sotalol (195 patients) or dofetilide (184 patients). Among these patients, 110 (56.4%) in the sotalol group and 111 (58.4%) in the dofetilide group received an initial dose that exceeded the recommended dose based on either their estimated creatinine clearance, QTc, or both. Hence, there was an increased probability of QTc prolongation observed with the use of nonstandard dosages of sotalol and dofetilide when compared to standard doses. However, the prescribing practices for these two drugs at the medical center did not demonstrate a preference for either medication over the other.

Procainamide

Procainamide is an antiarrhythmic medication of Class IA that operates by sodium channel blockade [21]. Some of its metabolites, such as N-acetylprocainamide (NAPA), have properties of blocking potassium channels, which may potentially result in the lengthening of the QT interval in individuals. Around 60% of procainamide is subjected to hepatic metabolism through N-acetyltransferase II, resulting in the formation of NAPA, whereas the remaining 40% is eliminated through renal pathways [22].

According to a case report on a male patient who was 65 years old and receiving hemodialysis treatment, who had prolonged QT intervals due to the procainamide metabolite NAPA. The patient had been diagnosed with hypertrophic obstructive cardiomyopathy (HOCM) after experiencing dyspnea on exertion following cardiac surgery a year prior and was being treated with carvedilol. However, due to persistent fatigue and shortness of breath, the patient was started on a type 1A Na channel blocker (750 mg/day of procainamide). A month later, the patient's ECG showed a prolonged QT interval with QTc intervals measuring 523 ms, which were previously normal. Procainamide was reduced from 750 mg to 500 mg per day as a consequence. The following day, the patient experienced cardiopulmonary arrest (CPA) and was resuscitated with DC shock. Blood tests revealed elevated levels of procainamide and NAPA, with NAPA levels exceeding the recommended threshold by 27.7 mg/mL. Consequently, procainamide administration was stopped. Therefore, to conclude, the QT prolongation was not caused by normal levels of procainamide but by toxic concentrations of NAPA [23].

Quinidine

Quinidine is a type of class 1A anti-arrhythmic medication that is commonly used for treating certain types of arrhythmias and malaria. Yet the common side effect of quinidine is TdP during the treatment of atrial fibrillation. Therefore, its usage for the treatment of AF is no longer recommended [24-27]. However, the Centers for Disease Control and Prevention (CDC) advises using higher doses of injectable (parenteral) quinidine gluconate in the treatment of falciparum malaria-resistant babesiosis infections - double to triple doses are recommended than used in AF [28].

This series of cases assessed the likelihood of QT interval prolongation and TdP in patients who were given the CDC-recommended dosing regimens of quinidine gluconate to treat resistant malaria or babesiosis. As per CDC, quinidine gluconate should be administered in the loading dose of 10 mg/kg over one to two hours, followed by either a continuous infusion of 0.02 mg/kg/min or an eight-hour interval. Further investigation is necessary to explore these risks and assess the safety of quinidine gluconate treatment.

In a wide metropolitan hospital network from January 2004 to December 2010, health records of patients who received intravenous quinidine to treat acute malaria or babesiosis were retrospectively examined. The purpose of the analysis was to gather more information regarding the use and efficacy of intravenous quinidine in treating these conditions. The research involved six participants who were treated with intravenous quinidine for either resistant malaria (in five cases) or babesiosis (in one case). These patients were continuously monitored using ECG at the bedside. Of the six patients, four exhibited QT interval prolongation (defined as either an increase in QTc interval of ≥60 ms from baseline or a total prolongation of ≥500 ms) and two instances of TdP were observed in experienced individuals.. The results indicate that there may be a possible risk linked with the use of quinidine for managing malaria or babesiosis, underscoring the necessity for additional research in this domain [29]. A noteworthy finding from this series of cases is that the highest QTc interval was measured in every patient during the initial 24 hours of receiving quinidine treatment. In addition, patients who experienced TdP or QTc interval prolongation had various risk factors for TdP, including imbalances in electrolyte levels and coexisting medical conditions like heart failure. Based on the results, it is recommended that patients who are treated with quinidine be monitored closely for possible QTc interval prolongation or TdP, especially during the initial 24 hours of therapy and in those with pre-existing risk factors. Additional research is required to verify and build upon these findings [30].

Flecainide

Flecainide is an antiarrhythmic drug classified as a class IC agent, commonly used for achieving sinus rhythm in individuals with supraventricular tachycardias or atrial fibrillation [31]. Takotsubo cardiomyopathy is a form of reversible cardiomyopathy that is usually characterized by the ballooning of the left ventricle apex and does not involve significant coronary arterial disease. This condition is uncommon and often occurs following episodes of extreme emotional stress, although it has also been associated with certain drugs such as catecholamines and antiarrhythmic medications like flecainide [32].

A case study conducted by Bodziock et al. in 2019 detailed a patient who developed long QT syndrome and Takotsubo cardiomyopathy occurred shortly after experiencing a life-threatening flecainide overdose [33]. The patient's serum flecainide levels were discovered to be more than three times the therapeutic dose two days following ingestion. This case report suggests that there might be a possible association between elevated doses of flecainide and a heightened risk of long QT syndrome and Takotsubo cardiomyopathy. Additional research is necessary to corroborate and extend these findings [33]. Table 3 shows the effectiveness of various drug therapies in managing cardiac arrhythmias.

**Table 3:** A summary of comparative studies on the effectiveness of various drug therapies in managing cardiac arrhythmias

Author	Year	Type of study	Patients	Purpose of study	Result
Takahashi et al. [14]	2021	Case Report	1	Efficacy of landiolol administration in the management of Torsades de pointes (TdP) induced by amiodarone, without the need for discontinuing the drug	After administration of amiodarone, a prolonged QT interval may develop, leading to the development of Torsades de pointes (TdP). However, it has been observed that intravenous administration of landiolol may help suppress TdP without the need to discontinue the use of amiodarone
Mourad et al. [15]	2019	Comparative Observational Study	46	To assess the degree of QT prolongation in patients who were being treated simultaneously with voriconazole and amiodarone	The co-administration of amiodarone and voriconazole led to a notable increase in the degree of QTc interval prolongation in comparison to that seen with either drug alone
Ting et al. [20]	2020	Retrospective Observational Study	379	The study's objectives were to review Dofetilide and Sotalol prescribing practises and compare the safety results of standard and unconventional dosing	The administration of initial doses of sotalol and dofetilide that exceed the recommended levels was connected to a higher frequency of QTc prolongation and a greater need for therapeutic adjustments
Ashida et al. [22]	2015	Case Report	1	Procainamide administration carries a significant risk of QTc prolongation in patients undergoing hemodialysis	Patients undergoing hemodialysis are susceptible to the build-up of higher levels of NAPA, a metabolite of procainamide, which could result in prolonged QTc intervals, even if their serum procainamide levels are normal
Wroblewski et al. [28]	2012	Case series	6	To evaluate the association between the recommended doses of Quinidine for malaria treatment and the risk of Torsades de Pointes (Tdp)	Using Quinidine at recommended doses was correlated with a higher risk of torsades de pointes (TdP)
Bodziock et al. [33]	2019	Case Report	1	To investigate the possible link between flecainide overdose and Takotsubo cardiomyopathy	A correlation observed between elevated doses of flecainide and a higher incidence of both long QT syndrome and Takotsubo cardiomyopathy

Limitations of the Study

The study had a limitation of a small sample size, only six studies were selected out of an initial pool of 68.

## Conclusions

QT prolongation can be a side effect of several antiarrhythmic drugs, such as amiodarone, sotalol, dofetilide, procainamide, quinidine, and flecainide. These proarrhythmic effects are linked with high doses, risk factors, concomitant diseases, and electrolyte imbalance. QT prolongation by these drugs can lead to torsade de pointes, which can be prevented or managed by dose reduction or withdrawal of the antiarrhythmic and sometimes by the use of a selective beta blocker. This topic needs further exploration and requires more clinical studies to be reported for a better understanding of the prevention and management of QT prolongation with the use of antiarrhythmic drugs.

## References

[1] Drew BJ, Ackerman MJ, Funk M (2010). Prevention of torsade de pointes in hospital settings: a scientific statement from the American Heart Association and the American College of Cardiology Foundation. Circulation.

[2] Al-Akchar M, Siddique MS (2022). Long QT Syndrome. StatPearls Publishing.

[3] Tang JK, Bennett MT, Rabkin SW (2019). Assessment of QT interval in ventricular paced rhythm: derivation of a novel formula. J Electrocardiol.

[4] Giudicessi JR, Roden DM, Wilde AA, Ackerman MJ (2018). Classification and reporting of potentially proarrhythmic common genetic variation in long QT syndrome genetic testing. Circulation.

[5] Beach SR, Celano CM, Sugrue AM, Adams C, Ackerman MJ, Noseworthy PA, Huffman JC (2018). QT prolongation, torsades de pointes, and psychotropic medications: a 5-year update. Psychosomatics.

[6] Uvelin A, Pejaković J, Mijatović V (2017). Acquired prolongation of QT interval as a risk factor for torsade de pointes ventricular tachycardia: a narrative review for the anesthesiologist and intensivist. J Anesth.

[7] Page MJ, McKenzie JE, Bossuyt PM (2021). The PRISMA 2020 statement: an updated guideline for reporting systematic reviews. BMJ.

[8] Vandenberk B, Vandael E, Robyns T (2016). Which QT correction formulae to use for QT monitoring?. J Am Heart Assoc.

[9] Recanatini M, Poluzzi E, Masetti M, Cavalli A, De Ponti F (2005). QT prolongation through hERG K(+) channel blockade: current knowledge and strategies for the early prediction during drug development. Med Res Rev.

[10] Thomas SH, Behr ER (2016). Pharmacological treatment of acquired QT prolongation and torsades de pointes. Br J Clin Pharmacol.

[11] Torres V, Tepper D, Flowers D (1986). QT prolongation and the antiarrhythmic efficacy of amiodarone. J Am Coll Cardiol.

[12] Zimetbaum P (2012). Antiarrhythmic drug therapy for atrial fibrillation. Circulation.

[13] Colunga Biancatelli RM, Congedo V, Calvosa L, Ciacciarelli M, Polidoro A, Iuliano L (2019). Adverse reactions of amiodarone. J Geriatr Cardiol.

[14] Takahashi K, Yamashita M, Sakaue T (2021). Suppression of amiodarone-induced torsade de pointes by landiolol in a patient with atrial fibrillation-mediated cardiomyopathy. Ann Noninvasive Electrocardiol.

[15] Mourad A, Stiber JA, Perfect JR, Johnson MD (2019). Real-world implications of QT prolongation in patients receiving voriconazole and amiodarone. J Antimicrob Chemother.

[16] Brüggemann RJ, Alffenaar JW, Blijlevens NM, Billaud EM, Kosterink JG, Verweij PE, Burger DM (2009). Clinical relevance of the pharmacokinetic interactions of azole antifungal drugs with other coadministered agents. Clin Infect Dis.

[17] Meierhenrich R, Helguera ME, Kidwell GA, Tebbe U (1997). Influence of amiodarone on QT dispersion in patients with life-threatening ventricular arrhythmias and clinical outcome. Int J Cardiol.

[18] Gueta I, Loebstein R, Markovits N, Kamari Y, Halkin H, Livni G, Yarden-Bilavsky H (2017). Voriconazole-induced QT prolongation among hemato-oncologic patients: clinical characteristics and risk factors. Eur J Clin Pharmacol.

[19] Weeke P, Delaney J, Mosley JD (2013). QT variability during initial exposure to sotalol: experience based on a large electronic medical record. Europace.

[20] Ting C, Malloy R, Knowles D (2020). Assessment of sotalol and dofetilide dosing at a large academic medical center. J Cardiovasc Pharmacol Ther.

[21] Mohamed AN, Abdelhady AM, Spencer D, Sowinski KM, Tisdale JE, Overholser BR (2013). Pharmacokinetic modeling and simulation of procainamide and N-acetylprocainamide in a patient receiving continuous renal replacement therapy: a novel approach to guide renal dose adjustments. Am J Kidney Dis.

[22] Ashida K, Mine T, Kodani T, Kishima H, Masuyama T (2015). Long QT syndrome caused by N-acetyl procainamide in a patient on hemodialysis. J Cardiol Cases.

[23] Somani R, Krahn AD, Healey JS (2014). Procainamide infusion in the evaluation of unexplained cardiac arrest: from the Cardiac Arrest Survivors with Preserved Ejection Fraction Registry (CASPER). Heart Rhythm.

[24] Camm AJ, Lip GY, De Caterina R (2012). 2012 focused update of the ESC Guidelines for the management of atrial fibrillation: an update of the 2010 ESC Guidelines for the management of atrial fibrillation. Developed with the special contribution of the European Heart Rhythm Association. Eur Heart J.

[25] Fuster V, Rydén LE, Asinger RW (2001). ACC/AHA/ESC Guidelines for the Management of Patients With Atrial Fibrillation: Executive Summary A Report of the American College of Cardiology/American Heart Association Task Force on Practice Guidelines and the European Society of Cardiology Committee for Practice Guidelines and Policy Conferences (Committee to Develop Guidelines for the Management of Patients With Atrial Fibrillation) Developed in Collaboration With the North American Society of Pacing and Electrophysiology. Circulation.

[26] Wann LS, Curtis AB, January CT (2011). 2011 ACCF/AHA/HRS focused update on the management of patients with atrial fibrillation (updating the 2006 guideline): a report of the American College of Cardiology Foundation/American Heart Association Task Force on Practice Guidelines. Circulation.

[27] Allen LaPointe NM, Li P (2000). Continuous intravenous quinidine infusion for the treatment of atrial fibrillation or flutter: a case series. Am Heart J.

[28] Wroblewski HA, Kovacs RJ, Kingery JR, Overholser BR, Tisdale JE (2012). High risk of QT interval prolongation and torsades de pointes associated with intravenous quinidine used for treatment of resistant malaria or babesiosis. Antimicrob Agents Chemother.

[29] Tisdale JE (2016). Drug-induced QT interval prolongation and torsades de pointes: role of the pharmacist in risk assessment, prevention and management. Can Pharm J (Ott).

[30] Andrikopoulos GK, Pastromas S, Tzeis S (2015). Flecainide: current status and perspectives in arrhythmia management. World J Cardiol.

[31] Dastidar AG, Frontera A, Palazzuoli A, Bucciarelli-Ducci C (2015). TakoTsubo cardiomyopathy: unravelling the malignant consequences of a benign disease with cardiac magnetic resonance. Heart Fail Rev.

[32] Gabriel L, Chenu P, Guédès A (2011). A possible association between takotsubo cardiomyopathy and treatment with flecainide. Int J Cardiol.

[33] Bodziock G, Armstrong C, Montgomery J (2019). Flecainide overdose presenting with long QT and acute Takotsubo cardiomyopathy. J Electrocardiol.

